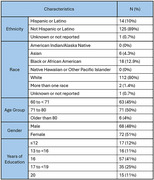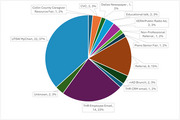# Lessons Learned in Study Recruitment from a Randomized Controlled Clinical Trial for Alzheimer's Disease Prevention

**DOI:** 10.1002/alz70860_102995

**Published:** 2025-12-23

**Authors:** Margaret McGregor, Jordan Zaenglein, Solymar Rivera‐Torres, Anna Tomlinson, Christopher Reagin, Tristyn Hall, Rong Zhang

**Affiliations:** ^1^ Institute for Exercise and Environmental Medicine, Texas Health Presbyterian Hospital Dallas, Dallas, TX, USA; ^2^ University of Texas Southwestern Medical Center, Dallas, TX, USA

## Abstract

**Background:**

The Impact of Intensive Treatment of Systolic Blood Pressure on Brain Perfusion, Amyloid, and Tau in Older Adults (IPAT Study, R01AG076660) is a randomized, controlled, clinical trial being conducted to determine the effects of intensive blood pressure treatment on brain amyloid and tau measured with positron emission tomography (PET) in older adults with hypertension. IPAT plans to enroll 180 participants with hypertension (SBP > 130 mmHg), age 60‐85, and randomize these participants into Usual Care (UC) or Intensive Blood Pressure Treatment (IT) group (NCT05331144). Our objective is to evaluate IPAT study recruitment strategies to understand the most effective approaches for study enrollment and the primary exclusion reasons for individuals interested in the study.

**Methods:**

The main recruitment strategies employed in the IPAT are: 1) affiliated hospital system databases (MyChart), 2) hospital employee and consumer marketing e‐mails, and 3) community outreach events such as educational talks and senior health fairs.

**Results:**

The study enrollment yields from various recruitment strategies are presented in Figure 1. In the first 2 years of the IPAT Study, from October of 2022 to November of 2024, 1735 individuals were phone screened and 140 were consented. The primary reasons for phone screening exclusion were: 1) SBP was <130 mmHg (*n* = 390), and 2) lack of interest from participants after opting to be contacted (*n* = 254). The primary reasons for participation halting post‐consent were: 1) SBP < 130 mmHg (*n* = 13), and 2) were unable to undergo an MRI or PET/CT scan (*n* = 7). Participant's demographics are presented in Table 1. Of note, among the consented, 80% are Non‐Hispanic White and 88% have a higher education level (≥ 13 years).

**Conclusion:**

Recruitment from hospital databases yielded the largest randomization rate of 31%. The primary reason for phone screening and post‐consent exclusion is SBP <130 mmHg, which suggests that the current recruitment strategies likely have reached to a population with normal or well‐controlled blood pressure. Thus, IPAT enrollment should consider recruiting from underserved populations who have limited access to blood pressure management such as from the socioeconomically disadvantaged minority groups or from rural communities.